# Supplementary material for: Exploring private land conservation non-adopters’ attendance at outreach events in the Chesapeake Bay watershed, USA
Source: PeerJ. 2021 Aug 31;9:e11959. doi: 10.7717/peerj.11959 (PMC8415281; doi:10.7717/peerj.11959)
Supplement: Supplemental Information 1 [file peerj-09-11959-s001.docx]

**Supplementary Material for:**

EXPLORING PRIVATE LAND CONSERVATION NON-ADOPTERS’ ATTENDANCE AT OUTREACH EVENTS IN THE CHESAPEAKE BAY WATERSHED, USA

**1. List of public directories used to create sampling frame**

Anne Arundel Economic Development Corp.

https://www.aaedc.org/agriculture/farm-business-profiles/

Baltimore County Farm Tour Map

https://marylandagriculture.org/wp-content/uploads/2016/04/Baltimore-County-Farm-Tour-Map-30-farms.pdf

Calvert County Agriculture

https://calvertag.com/185/Local-Products-Services

Carroll County Grown

https://carrollgrown.org/producer-list/

Cecil County Agriculture Directory

https://www.ccgov.org/government/economic-development/economic-development/agriculture/agriculture-directory

Charles County Agriculture Operating Farms Listings

http://www.charlescounty.org/webdocs/tourism/forms/agriculture.operating%20farms%20listing.pdf

Eastern Shore Harvest Directory

https://harvestdirectory.org/

Garrett County Local Food & Farm Guide

http://www.gcedonline.com/resources/garrettfarms/2017guide/mobile/index.html#p=1

Harford County Farm Finder

https://harfordgis.maps.arcgis.com/apps/webappviewer/index.html?id=d0dfc9c12bc447b8a049a0dbb2cf0ef2

Homegrown Frederick

http://www.homegrownfrederick.com/farm-directory

Howard County Economic Development Authority

http://www.hceda.org/farm-agriculture/locations/farm-search/

Maryland’s Best

https://marylandsbest.maryland.gov/

Maryland's Best 2020 Expo

https://marylandsbest.maryland.gov/wp-content/uploads/MBestExpoDirectory_web3-20.pdf

Queen Anne’s County Resource Directory

https://www.qac.org/BusinessDirectoryII.aspx?lngBusinessCategoryID=35

Southern Maryland Agricultural Development Commission Farm Guide

https://smadc.com/wp-content/uploads/2019/07/FarmGuideWEB.pdf\

**2. Email and Phone Scripts**

***First Email Attempt***

Dear Ms. / Mr. __________,

My name is _________, I’m a researcher at the University of Maryland. I'm writing because I'd like to set up a phone interview with you as a part of a project I am working on to improve outreach and technical assistance to farmers regarding best management practices. I’d be interested to have about a half-hour conversation with you about any outreach events you’ve been to or any interactions you have had with technical assistance, in order to understand what you think is working and what needs to be improved.

If you’re interested in talking with me, let me know when you’d like to talk and how best to reach you. My schedule is generally flexible, and I can likely accommodate whatever works for you, including mornings, afternoons, or evenings. And if you have any questions, don't hesitate to ask.

Thanks in advance for your time, and I look forward to hearing from you soon.

Best,

______

***Second Email Attempt***

Dear Ms. / Mr. _______,

I just wanted to follow-up on my email from last week and ask if you might be available for a phone interview to talk about your interactions with technical assistance regarding best management practices. See my previous email for more details. My schedule is generally flexible, and I can accommodate mornings, afternoons, or evenings. If you're interested in talking with me, I'd love to hear from you.

Thanks very much for your time.

Best,

_________

***First Phone Attempt***

*FOR ANSWERING MACHINE*

Hi, my name is ________. I’m a researcher at the University of Maryland calling for ____. I’m working on a project to improve outreach and technical assistance to farmers regarding best management practices, and I was wondering if you could help me out. I’d be interested to have about a half-hour conversation about events that you’ve been to or interactions with technical 58assistance that you’ve had to better understand what you think is working and what needs to improve. If you’re interested in participating in this sort of thing, feel free to call me back at 410-XXX-XXXX when you have the chance. Again, that’s 410-XXX-XXXX. Thanks very much. Bye.

*IF THEY ANSWER*

Hi, my name is ________. I’m a researcher at the University of Maryland calling for ______. I’m calling because I’m working on a project to improve outreach and technical assistance to farmers regarding best management practices. I’d be interested in talking to you about your experiences with outreach events or any interactions that you’ve had with technical assistance to better understand what you think is working and what needs to be improved. I anticipate that the conversation would be about a half an hour. Would this be something you’d be interested in?

***Second Phone Attempt***

*FOR ANSWERING MACHINE*

Hi, my name is ________. I’m a researcher at the University of Maryland calling for ____. I’m calling to follow-up on a message I left you last week. I’m working on a project to improve outreach and technical assistance to farmers regarding best management practices, and I was wondering if you could help me out. If you’d be interested in having a half-hour conversation about your experiences and thoughts on improvement, please feel free to call me back at 410-XXX-XXXX when you have the chance. Again, that’s 410-XXX-XXXX. Thanks very much. Bye.

*IF THEY ANSWER*

Same as above.

**3. Questionnaire with codebook**

*BMP = Best management practice, a more common term for agricultural conservation practice*

***SECTION 1: FARM/FARMER CHARACTERISTICS***

***1. Since this is our first time talking, it would be helpful for me to hear a little about your farming operation. How long have you worked this land, what do you produce, how many acres, and how much do you own vs. rent? And then we can get into some of the specifics.***

***1a. In production right now, or as part of a rotation (for animals, write in number and if known, whether pastured confined):***

__ Beef cattle

__ Dairy cattle

__ Goats

__ Sheep

__ Pigs

__ Poultry

__ Eggs

__ Vegetables

__ Other grains

__ Corn

__ Soybeans

__ Hay

__ Other, explain ________________

***1b. Total acres either in production or fallow:***

__

***1c. Years working this land?***

___

***1d. Tenure:***

__ Non-operating owner

__ Operating owner

__ Non-owner operator (leased land)

__ Own some, rent some

***2. What kinds of best management practices do you use on the farm? [Follow up by asking about any others that are on the list below]***

ANIMALS

__ Stream exclusion fencing

__ Rotational grazing/pasture management

__ Manure storage facility

CROPS

__ No-till

__ Cover crops

__ Contour plowing

__ Crop rotation

__ Variable rate application

WATER

__ Forested riparian buffers

__ Grassed riparian buffers

__ Filter strips

__ Grassed waterways

__ Other, explain _________________

***SECTION 2: OUTREACH EVENTS***

***3a. I am interested to know more about any outreach events focused on agricultural land management that you may have attended. Have you ever attended any outreach event that discusses agricultural land management?***

__ Yes

__ No

***3b. In 2019, about how often did you attend events that discussed agricultural land management?***

__ More than once a month

__ Once a month

__ Once every few months

__ Once in 2019

__ Never in 2019

[If yes, ask following questions. If no, go to question 21]

***4. If any, how many of these discussed any of the best management practices listed above?***

___

*[If they attended an event that discussed BMPs, include the language listed in the brackets]*

***5. Can you tell me about the most recent event you attended [that discussed one of the BMPs you mentioned]? When was it, what was it about, who organized it?***

***5a. When it was?***

__ Don’t remember

_____ Month/season and year

***5b. Who organized it?***

__ Don’t remember

__ NRCS

__ SWCD

_________ Non-profit (which one)

_________ Private group (which one)

__ University extension

__ County extension

______ Other (what?)

***5c. What it was about:***

_____________

***[5d. What BMP was discussed at the event]:***

_____________ (select from list above, or write multiple if applicable)

***6a. Was this event your first time learning about the topic [or BMP]?***

__ Don’t remember

__ Yes

__ No

***6b. If no, from where had you learned about this topic [BMP] before?***

__ Already does this practice

__ Knows of others who do it or have done it

__ Searched for information about this practice

__ Received information about this practice

__ Don’t remember

***7. How did you first hear about the event? [If person was an organizer, check that box and then check boxes for how they advertised it]***

__ Don’t remember

__ Was an organizer

CHANNEL

__ Email

__ Social media

__ Mail

__ Flyer

SOURCE

__ Another farmer

__ A non-farming peer

__ A family member

__ NRCS

__ SWCD

__ Non-profit (which one)

__ University Extension

__ County Extension

__ Private group (which one)

__ Other, describe

__ Don’t remember

***8. Among the event advertisements you see, how do you filter between what to put in the trash and what to consider attending?***

*_______________*

***9. What motivated you to attend the event?***

__ Don’t remember

INCENTIVES

__ Certification credits

__ Food

LOGISTICS

__ It was virtual

__ It was nearby

__ It didn’t cost too much

__ It wasn’t too long

__ I didn’t have anything better to do at the time

__ I wanted to get out of the house/off the farm

SOCIAL INFLUENCE

__ I know the organizers

__ I didn’t feel pressured into doing anything

__ I knew someone else going

__ My family wanted me to go

__ A trusted source told me it would be worthwhile

CONTENT

__ The subject matter, describe

__ An interesting guest speaker

__ I was involved in organizing the event

__ Other, describe

***10. What information did you have about the event prior to attending it?***

__ Don’t remember

__ Date, time, place

__ Subject matter

__ Who was organizing

__ Who was going

__ Other

***11. Did you know others who were planning to attend ahead of time?***

__ No

__ Knew the organizer

__ Knew other farmers attending

__ Was a presenter

__ Don’t remember

***12. Did you have any questions about whether or not you should or could attend? If so, what were they?***

__ No

__ Don’t remember

LOGISTICS

__ Inconvenient time/date

__ Inconvenient place

__ Event cost too much money

SOCIAL INFLUENCE

__ Didn’t know others going

__ Didn’t trust information being provided by this source

__ Didn’t want to be pressured to change farm management

CONTENT

__ Unsure if topic applied to me

__ Other, describe

***13. What time of day was the event?***

__ Early morning

__ Late morning

__ Early afternoon

__ Late afternoon

__ Early evening

__ Late evening

__ Don’t remember

***14. How long was the event?***

__ < 1 hour

__ 1-2 hours

__ 2-4 hours

__All day

__ Multiple days

__ Don’t remember

***15. Was the event on a weekend or weekday?***

__ Weekend

__ Weekday

__ Don’t remember

***16. Was the event virtual or in-person?***

__ Virtual

__ In-person

***17. Did it cost money to attend the event? If so, how much?***

__ No

__ Yes, how much?

__ Don’t remember

***18. Did you seek additional information about the practices discussed after the event? If so, who?***

__ No

__ Don’t remember

__ Other attendees

__ Farmers who didn’t attend

__ The event organizers

__ Other experts on the practice

__ Other sources (internet, etc.)

***19. Did you share information about the practices discussed at the event with anyone after the event? If so, who?***

__ No

__ Don’t remember

__ Farmers who didn’t attend

__ Family

__ Social media

__ Other experts on the practice

***20. Any last questions for myself or anything that we didn’t touch on that you think I should know?***

Describe: _______________

[Questions for people who have not attended an outreach event]

***21. Ok, have you ever heard about any kind of agricultural land management outreach event being organized in your area? If so, for the most recent event you heard of, how did you hear about it?***

__ No

__ Don’t remember

CHANNELS

__ Email

__ Social media

__ Mail

__ Flyer

SOURCE

__ Another farmer

__ A non-farming peer

__ A family member

__ NRCS

__ SWCD

__ Non-profit (which one)

__ University Extension

__ County Extension

__ Private group (which one)

__ Other, describe

***22. Why did you decide not to attend that event?***

__ Don’t remember

LOGISTICS

__ Inconvenient time/date

__ Inconvenient place

__ Event cost too much money

SOCIAL INFLUENCE

__ Didn’t know others going

__ Didn’t trust information being provided by this source

__ Didn’t want to be pressured to change farm management

CONTENT

__ Unsure if topic applied to me

__ Other, describe

***23. Do you plan to attend any outreach events on agricultural land management in the future?***

__ Yes

__ No

__ Maybe

***24. What would motivate you to attend an outreach event on agricultural land management?***

__ Don’t know

INCENTIVES

__ Certification credits

__ Food

LOGISTICS

__ It was virtual

__ It was nearby

__ It didn’t cost too much

__ It wasn’t too long

__ I didn’t have anything better to do at the time

__ I wanted to get out of the house/off the farm

SOCIAL INFLUENCE

__ I know the organizers

__ I didn’t feel pressured into doing anything

__ I knew someone else going

__ My family wanted me to go

__ A trusted source told me it would be worthwhile

CONTENT

__ The subject matter, describe

__ An interesting guest speaker

__ I was involved in the event

__ Other, describe

***25. Any last questions for myself or anything that we didn’t touch on that you think I should know?***

Describe: _______________

**4. Summary statistics about respondents**

**
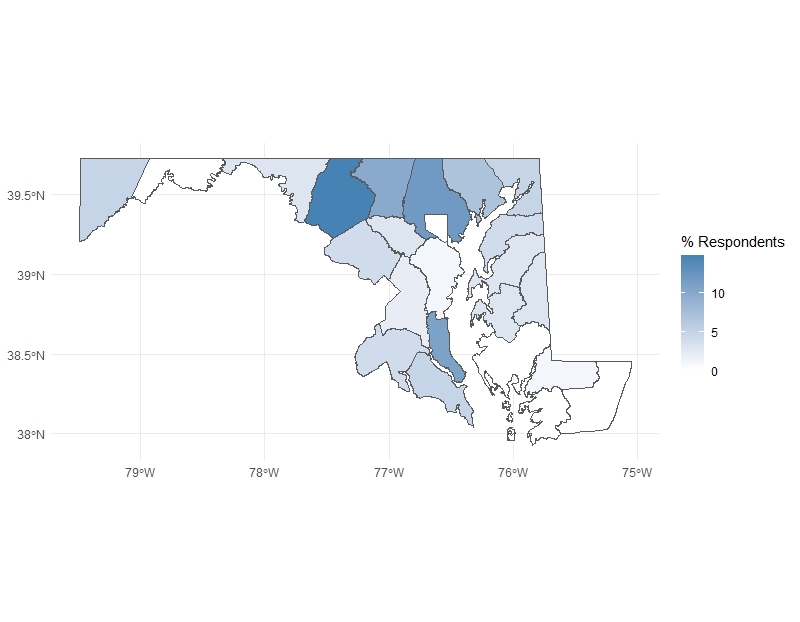
**

**Figure S1:** Map of Maryland showing percent of respondents per county

**Table S1:** Comparison of between 2017 Ag Census data and respondents from the phone interviews

|  | 2017 Maryland Ag Census | Our sample |
| --- | --- | --- |
| Size of farm (acres) | Percent of farms | Percent of farms |
| 1-9 | 18 | 14 |
| 10-49 | 37 | 26 |
| 50-179 | 27 | 30 |
| 180-499 | 11 | 19 |
| 500-999 | 4 | 7 |
| 1000-1999 | 2 | 5 |
